# Stable patients with suspected myocardial ischemia: comparison of machine-learning computed tomography-based fractional flow reserve and stress perfusion cardiovascular magnetic resonance imaging to detect myocardial ischemia

**DOI:** 10.1186/s12872-022-02467-2

**Published:** 2022-02-05

**Authors:** Dirk Lossnitzer, Selina Klenantz, Florian Andre, Johannes Goerich, U. Joseph Schoepf, Kyle L. Pazzo, Andre Sommer, Matthias Brado, Friedemann Gückel, Roman Sokiranski, Tobias Becher, Ibrahim Akin, Sebastian J. Buss, Stefan Baumann

**Affiliations:** 1 Department of Cardiology, Angiology and Pneumology, University of Heidelberg, Im Neuenheimer Feld 410, 69120 Heidelberg, Germany; 2 First Department of Medicine-Cardiology, University Medical Centre Mannheim, Mannheim, Germany; 3 DZHK (German Centre for Cardiovascular Research), Partner Site Heidelberg/Mannheim, Mannheim, Germany; 4 The Radiology Center, Sinsheim-Eberbach-Erbach-Walldorf-Heidelberg, Heidelberg, Germany; 5 Division of Cardiovascular Imaging, Department of Radiology and Radiological Science, Medical University of South Carolina, Charleston, SC USA

**Keywords:** Atherosclerosis, Cardiovascular magnetic resonance imaging, Coronary artery disease, Coronary CT angiography, Fractional flow reserve derived from coronary computed tomography angiography, Myocardial ischemia

## Abstract

**Background:**

Machine-Learning Computed Tomography-Based Fractional Flow Reserve (CT-FFR_ML_) is a novel tool for the assessment of hemodynamic relevance of coronary artery stenoses. We examined the diagnostic performance of CT-FFR_ML_ compared to stress perfusion cardiovascular magnetic resonance (CMR) and tested if there is an additional value of CT-FFR_ML_ over coronary computed tomography angiography (cCTA).

**Methods:**

Our retrospective analysis included 269 vessels in 141 patients (mean age 67 ± 9 years, 78% males) who underwent clinically indicated cCTA and subsequent stress perfusion CMR within a period of 2 months. CT-FFR_ML_ values were calculated from standard cCTA.

**Results:**

CT-FFR_ML_ revealed no hemodynamic significance in 79% of the patients having ≥ 50% stenosis in cCTA. Chi^2^ values for the statistical relationship between CT-FFR_ML_ and stress perfusion CMR was significant (*p* < 0.0001). CT-FFR_ML_ and cCTA (≥ 70% stenosis) provided a per patient sensitivity of 88% (95%CI 64–99%) and 59% (95%CI 33–82%); specificity of 90% (95%CI 84–95%) and 85% (95%CI 78–91%); positive predictive value of 56% (95%CI 42–69%) and 36% (95%CI 24–50%); negative predictive value of 98% (95%CI 94–100%) and 94% (95%CI 90–96%); accuracy of 90% (95%CI 84–94%) and 82% (95%CI 75–88%) when compared to stress perfusion CMR. The accuracy of cCTA (≥ 50% stenosis) was 19% (95%CI 13–27%). The AUCs were 0.89 for CT-FFR_ML_ and 0.74 for cCTA (≥ 70% stenosis) and therefore significantly different (*p* < 0.05).

**Conclusion:**

CT-FFR_ML_ compared to stress perfusion CMR as the reference standard shows high diagnostic power in the identification of patients with hemodynamically significant coronary artery stenosis. This could support the role of cCTA as gatekeeper for further downstream testing and may reduce the number of patients undergoing unnecessary invasive workup.

## Background

For many years, the efficacy of percutaneous coronary intervention (PCI) in the treatment of coronary artery disease (CAD) in chronic coronary syndrome was beyond dispute. However, several studies including DEFER have recently challenged this perception by showing futility or even harm of PCI in patients with CAD without hemodynamically significant stenosis. [1, 2] Current studies like ISCHEMIA even question the benefit of PCI in ischemia inducing stable CAD, except for the purpose of symptom relief in patients with angina. [3]

With regard to the ESC guidelines of 2019, non-invasive tests are therefore preferred over invasive coronary angiography (ICA) as the initial diagnostic modality for chronic coronary syndromes. [4] In the recent decade, coronary computed tomography angiography (cCTA) has established itself as a powerful tool for the non-invasive assessment of coronary stenosis, particularly in patients with low-to-intermediate pretest probability. [5, 6] Despite its high negative predictive value, it has long been recognized that cCTA correlates comparatively poorly with the actual hemodynamic significance of stenosis, and that cCTA overestimates stenosis severity. [7–9] Therefore ESC guidelines recommend further functional testing after cCTA unless the stenosis is severe (> 90% diameter stenosis, except left main stem > 50%). [4]

There exists a large variety of cardiac imaging techniques for this further testing. Stress perfusion cardiovascular magnetic resonance imaging (CMR) is an established functional non-invasive method to guide coronary revascularization. It enables the detection of myocardial ischemia based on the first-pass kinetics of gadolinium contrast agent after adenosin-induced vasodilatation. The MR-INFORM trial demonstrated prospectively the non-inferiority of stress perfusion CMR compared to invasive fractional flow reserve (FFR) measurements with respect to major adverse cardiac events [10]. Despite its high diagnostic power reported in a number of studies, CMR also has limitations that prevent it from becoming the standard method of ischemia assessment. [11–14]

CT derived fractional flow reserve (CT-FFR) is a new and promising non-invasive approach that combines morphological and functional information about coronary lesions in a single diagnostic tool. CT-FFR uses a patient's standard cCTA study along with a fluid dynamics model to calculate a value that can be interpreted similar than an invasive FFR measurement. Several studies and meta-analyses have shown that CT-FFR achieves better diagnostic accuracy than cCTA alone when compared to invasive FFR. [15–19] A more recent CT-FFR technology is based on a machine-learning algorithm (CT-FFR_ML_) that enables on-site use and reduces computation time. [20, 21] Furthermore, in contrast to stress perfusion CMR, CT-FFR_ML_ does neither require an additional image acquisition nor the application of a stress agent. Therefore, it is associated with lower costs, less procedural time, and fewer contraindications. [13]

The aim of this study was to compare the diagnostic performance of the novel functional non-invasive diagnostic tool CT-FFR_ML_ to standard cCTA in the detection of myocardial ischemia using stress perfusion CMR as reference standard.

## Methods

### Patient population and study design

Our study was designed as an observational, retrospective, single-center study (The Radiology Center, Sinsheim-Eberbach-Erbach-Walldorf-Heidelberg, Germany). The database query and patient inclusion were performed in a consecutive order. Each of our 141 included patients underwent a clinically indicated cCTA between January 2017 and September 2020, revealing at least one coronary artery stenosis with unclear hemodynamic relevance. In accordance with current ESC guidelines for chronic coronary syndromes, this finding was followed by a functional imaging test for myocardial ischemia. Therefore, our study population received a stress perfusion CMR within two months of the initial cCTA. In summary, our inclusion criteria comprise a clinically indicated cCTA showing one or more stenotic arteries with unclear hemodynamic relevance followed by a stress perfusion CMR without exceeding the interprocedural time of two months.

**Fig. 1:**
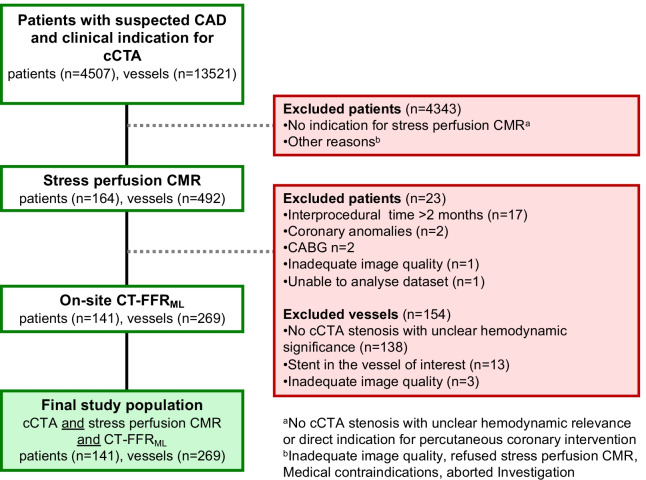
Flow chart. Development of study population. CT-FFR_ML_ = fractional flow reserve derived from coronary computed tomography angiography based on machine learning algorithm, cCTA = coronary computed tomography angiography, CMR = Cardiovascular magnetic resonance imaging, CABG = coronary artery bypass graft

We retrospectively analyzed cCTA images with on-site CT-FFR_ML_, if patients did not meet one of the following exclusion criteria: software problems with patients’ data, inadequate cCTA image quality, coronary anomalies, severe left main disease, severe stenosis at coronary ostia, coronary artery bypass grafts, aneurysms, chronic total occlusion or previous PCI in a vessel of interest (Fig. 1). Patient baseline characteristics and cardiovascular risk factors were obtained from medical records. The study was approved by the local ethics committee (S-108/2020 and 2020-882R) and was conducted in accordance with the Declaration of Helsinki.

## Acquisition and analysis of cCTA datasets

Acquisition of the cCTA datasets were performed using a 2 × 192-slice dual-source CT System (Somatom FORCE, Siemens Healthineers, Forchheim, Germany). First, each patient underwent a low-dose calcium scoring scan in order to obtain the Agatston score equivalent. [22] Prior to this scan, nitroglycerin and beta blocker were administered sublingually and intravenously, respectively, with a target heart rate of < 65/min. For the contrast agent enhanced cCTA scan, patients received 80 ml of iodinated contrast agent (Iomeron 400; Bracco Imaging S.p.A., Milan, Italy) in an antecubital vein using a power injector (Stellant D; Medrad, Warrendale, PA, USA) followed by administration of a saline chaser. Experienced radiologists and cardiologists subsequently analyzed the cCTA studies using a dedicated workstation (Syngo VB30A; Siemens Healthineers, Forchheim, Germany). Following Society of Cardiovascular Computed Tomography (SCCT) guidelines, the coronary tree was sectioned into 18 segments and CAD-RADS™ was determined. [23, 24] Additionally, two experienced radiologists and one cardiologist blinded to the clinical representation of the patients graded each segment with respect to the structure of the plaque (calcified, soft, mixed, high-risk) and severity of the putative stenosis (cannot be assessed, minimal 1–24%, mild 25–49%, moderate 50–69%, severe 70–94%, subtotal 95–99%) in sense of consensus interpretation. CAD-RADS™ ≥ 3 (≥ moderate 50–69%) as well as a segment severity grade of ≥ 50% define a positive cCTA result in this study. In case of any conflict consensus reading was performed.

## Acquisition and analysis of stress perfusion CMR dataset

Electrocardiogram-gated cine images were acquired using a segmented steady-state free precession (true-FISP) sequence. 4-, 2-, and 3-chamber views as well as sequential short-axis views (apical, midventricular, and basal) were acquired during end-expiratory breath-holds for anatomical and functional measurements on a 1.5 T magnetic resonance imaging (MRI) scanner (Aera, Siemens Healtineers, Forchheim, Germany). Electrocardiographic rhythm and blood pressure were monitored continuously. For perfusion imaging, intravenous adenosin was infused at 140 µg/kg/min for at least 3 min, with up-titration to 210 µg/kg/min, if there was failure to achieve the pre-specified physiological target i.e. patients’ hemodynamic responses were considered adequate if they demonstrated an increase in heart rate ≥ 10 bpm. Following this, a bolus of the 0.05 mmol/kg body weight gadobutrol (Gadovist, BayerVital, Germany) was administered intravenously. 3-short-axis slices of the left ventricle were acquired per cardiac cycle during free-breathing. Late gadolinium enhancement (LGE) imaging was performed ten minutes after injection of gadolinium contrast in continuous short-axis, 4-chamber 3-chamber and 2-chamber long-axis views using a phase sensitive inversion recovery (PSIR) 2D sequence. All images were analyzed by cardiologists and radiologists with consensus reading using the multi-modality 3D-enabled workstation (Syngo VB30A; Siemens Healthineers, Forchheim, Germany).

## Analysis of machine-learning computed tomographic-based fractional flow reserve

**Fig. 2:**
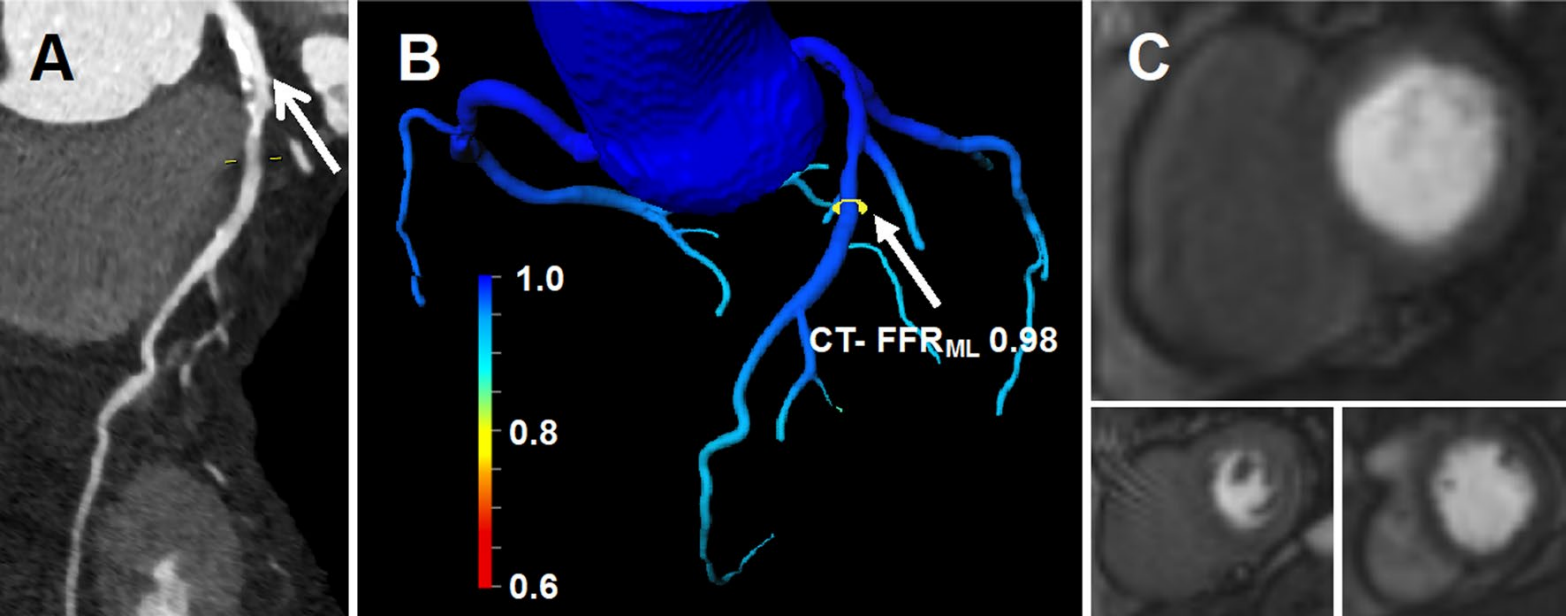
80-year-old male patient with pre-test probability of CAD of 37% (CAD consortium (%)- basic model). **a** cCTA demonstrates a high-risk plaque in the main stem of the left coronary artery and in the proximal left anterior descending coronary artery (arrow). **b** CT-FFR_ML_ software provides a color-coded 3-dimensional mesh of the coronary tree and calculated a value of 0.98 in an area distal of the stenosis (arrow). **c** Stress perfusion CMR in three short axis slices shows no perfusion deficit and thus no myocardial ischemia. CAD = coronary artery disease; cCTA = coronary CT angiography; CT-FFR_ML_ = Fractional flow reserve derived from coronary computed tomography angiography based on machine learning algorithm; CMR = cardiovascular magnetic resonance imaging

**Fig. 3:**
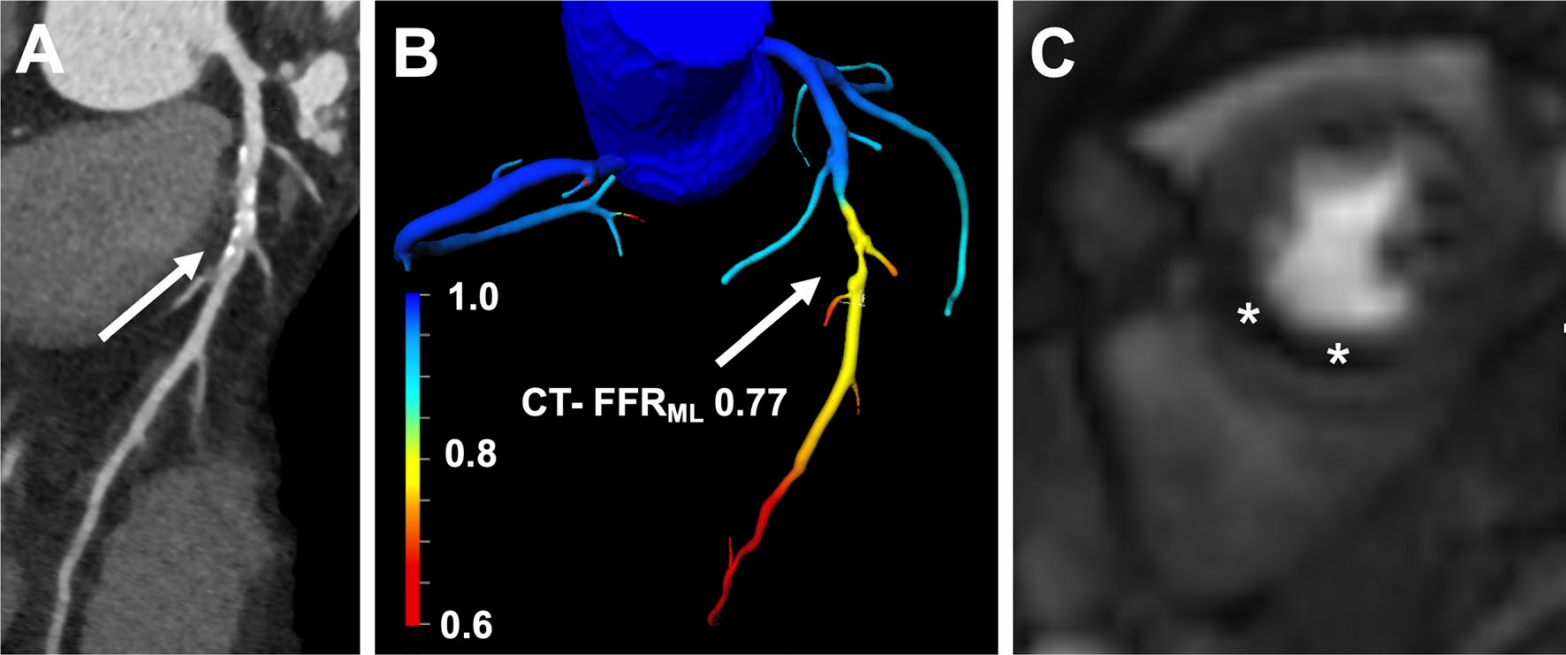
53-year-old male patient with suspected CAD and arterial hypertension. **a** cCTA illustrates a moderately graded stenosis (50–69%) with unclear hemodynamic relevance caused by mixed structured plaques in the mid left anterior descending coronary artery (arrow). **b** Color-coded 3-dimensional mesh created by CT-FFR_ML_ software shows a flow-limiting stenosis with a measured value of 0.77 (arrow). **c** a midventricular short axis stress perfusion CMR image demonstrates a significant perfusion deficit in the left anterior descending coronary artery territory, which correlates with the myocardium subtended by the hemodynamically significant lesion identified on CT-FFRML; CAD = coronary artery disease; cCTA = coronary CT angiography; CT-FFR_ML_ = Fractional flow reserve derived from coronary computed tomography angiography based on machine learning algorithm; CMR = cardiovascular magnetic resonance imaging

For calculation of the fractional flow reserve derived from the cCTA dataset of our patients, we used an on-site CT-FFR_ML_ prototype installed on a conventional workstation (Syngo VB30A; Siemens Healthineers Forchheim, Germany). This prototype is based on a machine-learning algorithm (Siemens cFFR, version 3.2; Siemens Healthineers, Forchheim, Germany), which is not yet commercially available. First, the software proposes manually adjustable centerlines that run through the lumen of each detected coronary artery. Then, it calculates the boundaries of the vessels and excludes coronary plaques. After acceptance by the user a 3-dimensional color-coded mesh of the coronary tree and aortic root is generated (Figs. 2, 3). An estimated CT-FFR value is provided for every spot on the mesh. Similar to interpreting invasive FFR results, a coronary stenosis with a CT-FFR value less than or equal to the cut-off value 0.80 is considered to be hemodynamically significant. [25] CT-FFR_ML_ measurements were performed directly distal of a stenosis. No CT-FFR_ML_ value was determined for vessels without at least one stenosis with unclear hemodynamic significance in cCTA.

## Statistical analysis

Categorial data is given as counts and proportions and continuous data as mean ± standard deviation. Diagnostic test evaluation was performed using Receiver Operating Characteristic Curve analyses, including Area Under the Curve calculations. Logistic regression models were applied to assess the diagnostic power of the CT-derived parameters. A p-value < 0.05 was regarded as statistically significant. The sample size calculation was performed for the AUC analysis of the CT-FFR_ML_. We hypothesized an AUC of 0.75 for CT-FFR_ML_ and defined the null hypothesis value as 0.5. As CMR perfusion was the reference standard, the ratio of negative to positive cases was 124/17. With an alpha value of 0.05 and a beta value of 0.2, resulting in a power of 0.8, the needed sample size was 100 cases (12 positive). An interobserver variability was calculated using Cohen’s Kappa statistic. Statistical analyses were conducted using dedicated statistical software (Medcalc 19.6, MedCalc Software Ltd, Ostend, Belgium).

## Results

Between January 2017 and September 2020, 4507 patients received a clinically indicated cCTA examination. Stress perfusion CMR was performed in 164 patients showing a CAD with at least one stenosis of unclear hemodynamic significance.17 cases were excluded, because the interprocedural time of 2 months between cCTA and CMR was exceeded. Furthermore, coronary anomalies, CABG (coronary artery bypass graft), inadequate image quality or software problems prevented successful CT-FFR_ML_ analysis in a total of 6 cases. Our final study population included 141 patients. After the exclusion of 3 vessels due to insufficient image quality and 13 vessels due to stents, cCTA and stress perfusion CMR images of 407 vessels (96%) were included in the final analysis. CT-FFR_ML_ was calculated in 269 vessels (64%) having stenosis with unclear hemodynamic relevance as prespecified in the study design (Fig. 1).

Baseline characteristics of the included patients are presented in Table 1. In our study population, we calculated the mean pretest probability of having CAD to be 20% ± 12% in the CAD consortium-basic model and the mean number of cardiovascular risk factors to be 2.0 ± 1.1. [26]

**Table 1:** Baseline characteristics

Parameter	Mean value ± standard deviation or frequency
Age (years)	67 ± 9
Men	110 (78%)
Body-mass-index (kg/m^2^)	28 ± 5
Systolic blood pressure (mmHg)	154 ± 20
Diastolic blood pressure (mmHg)	86 ± 11
Heart rate (beats per min)	65 ± 13
**Cardiovascular risk factors**	
Arterial hypertension^a^	77 (64%)
Family history for CAD	55 (45%)
Hypercholesterolemia^b^	47 (41%)
Diabetes mellitus	24 (20%)
Smoker	15 (12%)
Pre-test probability of CAD	20 ± 12
**Baseline blood values**	
Cholesterol (mg/dl)	188 ± 50
High-density lipoprotein (mg/dl)	52 ± 15
Low-density lipoprotein (mg/dl)	118 ± 42
Triglycerides (mg/dl)	158 ± 103
Hemoglobin A1c (%)	6.0 ± 0.9
Creatinine (mg/dl)	1.0 ± 0.2
**Baseline medication**	
Statin	63 (53%)
beta-blocker	39 (37%)
Aspirin	40 (34%)
Angiotensin-converting-enzyme inhibitor or AT_1_ receptor blocker	30 (32%)
Calcium channel blocker	13 (14%)
P2Y_12_ inhibitor	4 (3%)
Nitrates	3 (3%)

Unless otherwise specified, data are numbers of patients with percentage in parentheses. Data are mean ± standard deviation (SD). ^a^Defined as blood pressure >140 mmHg systolic, >90 mmHg diastolic, or use of an antihypertensive medication. ^b^Defined as a total cholesterol level of >200 mg/dL or use of lipid lowering medication. CAD = coronary artery disease, BMI = body mass index

Among all 141 patients, 129 (91%) patients had at least one diameter stenosis ≥ 50% in cCTA (positive cCTA result). Only 12% (16 of 129) of the cCTA positive cases showed a positive stress perfusion CMR demonstrating the hemodynamic relevance of the assessed stenosis. Hence, the functional CMR examination could prevent invasive coronary angiography in 88% of these patients (113 of 129). In 79% (102 of 129) of all cCTA positive patients, CT-FFR_ML_ alone could have revealed lesions without ischemia (true negative results) and thus, would have rendered further downstream testing as stress perfusion CMR unnecessary. This effect becomes more pronounced if only intermediately-graded stenosis (50–69%) in cCTA were considered (101 patients); this yields a percentage of 6% (6 of 101) positive stress perfusion CMR results, and 12% (12 of 101) positive CT-FFR_ML_ results. cCTA, CT-FFR_ML_ and stress perfusion CMR findings are presented in Table 2. We could calculate an interobserver kappa coefficient of 0.94 for our cCTA analyses which demonstrates a high interobserver agreement.

**Table 2:** Findings of cCTA, CT-FFR_ML_ and stress perfusion CMR

Parameter	Mean value ± standard deviation or frequency (%)
**cCTA**	
Agatston score^a^	657 ± 808
Agatston score interquartile range	759
No. of patients Agatston score > 400	74 (54%)
Luminal stenosis ≥ 50%	129 (91%)
Mean No. of vessels per patient	1.7 ± 0.9
Luminal stenosis ≥ 70%	28 (20%)
Mean No. of vessels per patient	0.3 ± 0.6
**CT-FFR**_**ML**_	
CT-FFR_ML_ ≤ 0.8	27 (19%)
**Stress perfusion CMR**	
Significant perfusion deficit^b^	17 (12%)

Unless otherwise specified, data are numbers of patient, with percentages in parentheses. Data are mean ± standard deviation (SD) or frequency (%). ^a^Agatston score measured in 138 patients. ^b^Defined as two neighboring slices or in midventricular or basal part more than 60 degrees or in apical part more than 90 degrees or a transmural defect irrespective of location. (37) cCTA = coronary CT angiography; CT-FFR_ML_ = fractional flow reserve derived from coronary computed tomography angiography based on machine learning algorithm; CMR = cardiovascular magnetic resonance imaging

Our univariate logistic regression analysis shows a highly significant relationship between CT-FFR_ML_ and stress perfusion CMR, with Chi^2^ = 47 (*p* < 0.0001). However, the relationship between cCTA in patients with stenosis ≥ 70% and stress perfusion CMR is significant as well, with Chi^2^ = 15 (*p* < 0.0001). In contrast, cCTA with ≥ 50% stenosis could not reach the level of significance upon Chi^2^-testing, and thus could not predict hemodynamic significance of stenosis assessed by stress perfusion CMR.

**Fig. 4:**
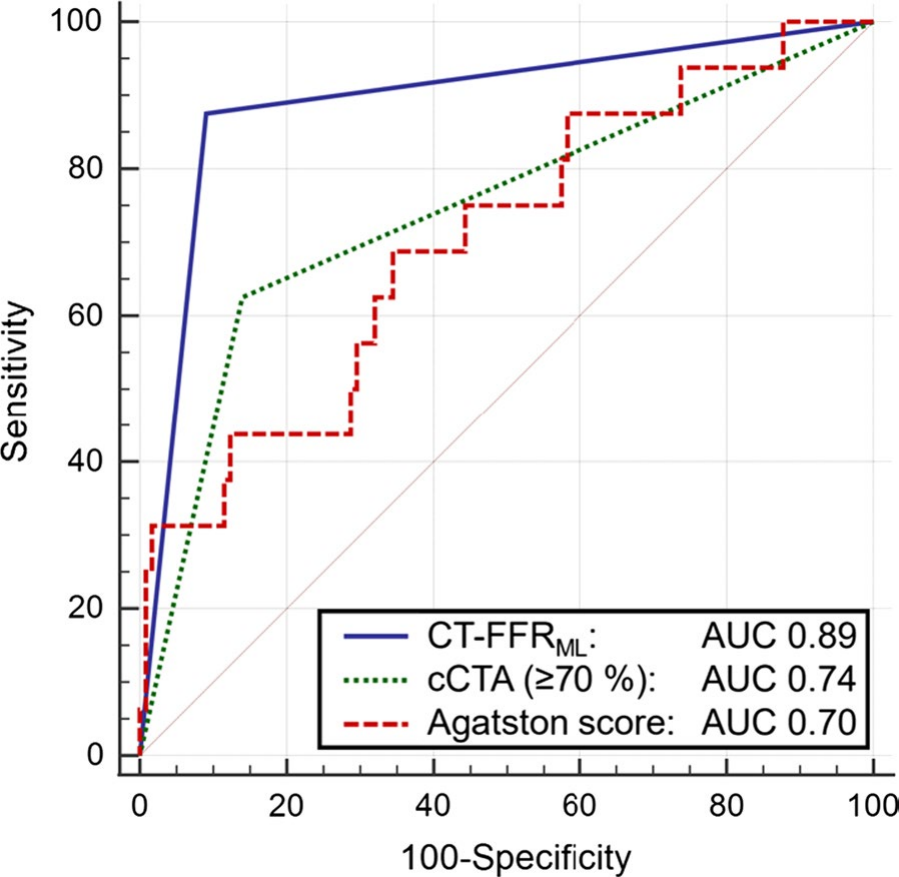
ROC of Agatston score, cCTA (stenosis ≥ 70%) and CT-FFR_ML_ with stress perfusion CMR as reference standard. The AUC for detection of ischemia inducing stenosis by CT-FFR_ML_ was 0.89. Agatston score and cCTA (stenosis ≥ 70%) provide AUC values of 0.70 and 0.74 (n = 138). CAD = coronary artery disease; cCTA = coronary CT angiography; CT-FFR_ML_ = Fractional flow reserve derived from coronary computed tomography angiography based on machine learning algorithm; CMR = cardiovascular magnetic resonance imaging

Using stress perfusion CMR as the reference standard, we calculated for CT-FFR_ML_ a per patient (n = 141) sensitivity of 88%, specificity of 90%, positive predictive value of 56%, negative predictive value of 98% and accuracy of 90%. The accuracy of cCTA alone to predict hemodynamically significant stenosis was 19% for cCTA (≥ 50%) and 82% for cCTA (≥ 70%) when compared to stress perfusion CMR. In addition to notably higher accuracies, the positive predictive values from cCTA to CT-FFR_ML_ were markedly improved as well (Table 3). As illustrated in Fig. 4, the diagnostic accuracy measured by the AUC of CT-FFR_ML_ (0.89) surpassed those of the Agatston score (0.70) as well as the degree of cCTA stenosis ≥ 70% (0.74) in detection of ischemia-inducing lesions with respect to the reference standard of stress perfusion CMR. Namely, there was a significant (*p* < 0.05) increase from 0.74 to 0.89 between cCTA ≥ 70% and CT-FFR_ML_.

**Table 3:** Diagnostic performance of CT-FFR_ML_, cCTA (≥ 50% stenosis) and cCTA (≥ 70% stenosis) using stress perfusion cardiovascular magnetic resonance imaging as reference standard

	cCTA (≥ 50%)	cCTA (≥ 70%)	CT-FFR_ML_ (≤ 0.80)
Sensitivity (%)	94 (71–100)	59 (33–82)	88 (64–99)
Specificity (%)	9 (5–15)	85 (78–91)	90 (84–95)
PPV (%)	12 (11–14)	36 (24–50)	56 (42–69)
NPV (%)	92 (60–99)	94 (90–96)	99 (94–100)
Accuracy (%)	19 (13–27)	82 (75–88)	90 (84–94)

CT-FFR_ML_ = fractional flow reserve derived from coronary computed tomography angiography based on machine learning algorithm; cCTA = coronary CT angiography; PPV = positive predictive value; NPV = negative predictive value

## Discussion

In this study, we conducted one of the first investigations into the diagnostic power of the recently introduced CT-FFR_ML_ software for the assessment of the hemodynamic relevance of coronary stenosis in comparison to the established diagnostic modality of stress perfusion CMR. We also examined, if there is an additional value that CT-FFR_ML_ provides over the standard cCTA diagnostic approach. Taken together, our data herein suggest high diagnostic power and notable potential in the clinical utility of CT-FFR_ML_ in the management of chronic CAD.

Although the Food and Drug Administration granted approval of the CT-FFR_ML_ in 2015, the software is currently only available for research purposes. To date, the only commercially available alternative is the fluid dynamics-based CT-FFR algorithm from HeartFlow® (HeartFlow Inc., Redwood, CA, USA) which is an off-site software. An increasing interest in CT-FFR technology has spurred more investigations comparing CT-FFR to invasive FFR as DISCOVER-FLOW, DeFACTO and NXT. These studies yielded values for sensitivity, specificity, accuracy and AUC of 84–89%, 61–86%, 73–84% and 0.79–0.93, respectively. [16–18] Not only CT-FFR but also other functional diagnostic tools for ischemia detection were compared to the reference standard invasive FFR. These included single-photon emission computed tomography (SPECT), stress echocardiography and stress perfusion CMR with reported sensitivities of 0.70 (0.59–0.80), 0.77 (0.61–0.88), 0.90 (0.75–0.97) and specificities of 0.78 (0.68–0.87), 0.75 (0.63–0.85), 0.94 (0.79–0.99). [11] These diagnostic values, as well as the MR-INFORM noninferiority clinical-effectiveness trial, implicate an outstanding diagnostic performance of stress perfusion CMR when compared to other functional diagnostic modalities. [10]

In our study, we performed a comparison between CT-FFR_ML_ and stress perfusion CMR, which served as reference standard. We calculated sensitivity, specificity, accuracy and AUC values for CT-FFR_ML_ of 88%, 90%, 90% and 0.89. Our results thus corroborate findings of previous studies. Although CT-FFR_ML_ has demonstrated to be a reliable diagnostic tool in detection of ischemia inducing coronary stenosis, there are still some limitations. First, there was a notable number of patients (7–12%) in previous studies in which cCTA data were not evaluable by CT-FFR. [27] Of note, the proportion of excluded patients (4%) in this study was below average. This could be attributed to the improved image quality attained by the use of a state-of-the-art dual-source CT scanner of the third generation. [28]

Although CT technology has undergone considerable improvements, patient radiation exposure is unavoidable. Furthermore, there are still more false negative cases in CT-FFR_ML_ than in stress perfusion CMR, when comparing the sensitivities of previous studies referred to invasive FFR. [11, 12, 20] In our study population of 141 patients, there were 2 cases with a negative CT-FFR_ML_ but positive stress perfusion CMR. In clinical practice, these false negative results could have had led to wrong diagnosis and subsequent therapy for the respective patients. Finally, standard cCTA as well as CT-FFR_ML_ unlike stress perfusion CMR are not able to detect microvascular disease. However, computed tomography-based diagnostic techniques have many advantages over stress perfusion CMR. Stress agents like adenosin are not required for imaging, which implies that patients are not exposed to potential adverse effects. Additionally, stress perfusion CMR is very time-consuming, whereas cCTA is a fast imaging method. Renker et al. gave a calculation time for on-site CT-FFR of 37.5 ± 13.8 min. [29] But for CT-FFR_ML_ an average duration of only 11 ± 2 min per analysis was demonstrated. [30] In addition, stress perfusion CMR is an expensive imaging tool with limited availability compared to cCTA. [13] Finally, there is substantial number of contraindications for CMR including older non MRI safe implanted pacemakers and other electronical implants or magnetizable objects that do rarely affect cCTA or CT-FFR_ML_.

In summary, cCTA alone or in combination with CT-FFR_ML_ exhibits better clinical practicability compared to stress perfusion CMR for the detection of significant coronary stenosis. Besides its feasibility, the 5-year follow-up of SCOT-HEART illustrated that cCTA is able to reduce mortality from CAD and nonfatal myocardial infarction, compared to a standard of care group. These benefits were attributed to a refinement in healthy lifestyle and preventive therapies, but not by altering PCI strategy. [31] The two landmark trials SCOT-HEART and PROMISE suggest a notable benefit of cCTA as initial diagnostic approach for patients with stable CAD [31, 32]. However, they also point to an improvement in patient selection for ICA or PCI when adding a functional element to an anatomical diagnostic strategy [33]

We can confirm this conclusion through our study results. As we demonstrated, in 88% of patients showing obstructive CAD (≥ 50% stenosis), stress perfusion CMR showed no significant ischemia in these subjects. If there had not been subsequent stress perfusion CMR after the initial cCTA, this may have resulted in unnecessary ICA or even PCI in all of these cases. Not only stress perfusion CMR, but also CT-FFR_ML_ proved to be able to reduce unnecessary invasive diagnostic examinations. This discriminatory benefit through CT-FFR_ML_ has also been reported in previous studies. [19, 34] In addition, the accuracy of 19% for cCTA (≥ 50% stenosis) and of 82% for cCTA (≥ 70% stenosis) reflect the limited capability of cCTA to assess the hemodynamic relevance of coronary stenosis. Due to a small number (n = 12) of cCTA negative patients in our study population, accuracy for cCTA (≥ 50% stenosis) lies beyond the average of previous studies. Nevertheless, DISCOVER-FLOW, DeFACTO and NXT, also provided low accuracy for cCTA (≥ 50% stenosis) of 53–64% in the prediction of hemodynamic relevance. CT-FFR_ML_ leads to a remarkable improvement in accuracy (up to 90%) in our study.

The calculated AUC depicts a moderate diagnostic value in detection of ischemia inducing coronary stenosis of Agatston score (0.70). This result is consistent with AUC of Agatston score referred to invasive FFR. [34] Superiority of the recent non-invasive diagnostic tool CT-FFR_ML_ to standard cCTA method related to the established stress perfusion CMR becomes particularly clear when considering the significant difference in already high AUC values (0.89 against 0.74).

All in all, our study is a further contribution to the long development of the non invasive diagnostic possibilities of chronic coronary heart disease. The combination of non invasive Agatston scoring, cCTA, CT-FFR_ML_ might prevent unnecessary invasive coronary angiographies in accordance to the current ESC guidelines when compared to the golden standard stress perfusion CMR. Recent studies demonstrated a significant correlation of CT-FFR_ML_ and morphological CT derived plaque parameters when compared with the reference standard RFR to detected hemodynamically significant coronary stenosis [35]. Furthermore CT-FFR_ML_ provided a good correlation with invasive iFR® measurements without vasodilator induced hyperemia [30]. Supplementary CT-FFR_ML_ might provide valuable guidance in necessary coronary interventions by a dedicated detection of ischemic myocardium especially in case of multiple equivalent coronary stenosis. The aim is to of find the most uncomplicated, precise and safe method for diagnosing hemodynamically relevant coronary stenoses in each and every individual patient.

## Limitations

When evaluating this study, some limitations should be considered. In first place, representativeness of our results for the general population is limited by our retrospective single-center study design and the relatively low number of included patients. Thus, small differences for instance the difference between the CTA and the Agatston score, which did not reach statistical significance in our population might, have in larger one. In consequence additional studies are needed to further investigate our findings.

Second, we did not investigate reproducibility of CT-FFR_ML_ analyses for this study. However, all MRI and CT-FFR_ML_-measurements were performed as consensus readings of at least one experienced cardiologist and one experienced radiologist. Furthermore, previous studies from our own group and others yield high interobserver correlation, also among operators of variable expertise [30, 35, 36].

Third, due to ESC guidelines and ethical aspects patients without stenosis of unclear hemodynamical significance did not undergo stress perfusion CMR. This led to a lack of stress perfusion CMR images of patients providing stenosis graded < 50% in cCTA. Accordingly, the informative value of our calculated accuracy for cCTA ≥ 50% stenosis is limited. Furthermore, the comparison to actual gold standard invasive FFR would have been very interesting.

## Conclusion

Our study is one of the first to show the high diagnostic accuracy of CT-FFR_ML_ compared to stress perfusion CMR. Furthermore, the diagnostic values herein demonstrate that CT-FFR_ML_ is superior to cCTA alone. Moreover, as our data shows, CT-FFR_ML_ is able to reduce unnecessary ICAs when it is added to the standard CT diagnostic algorithm.

## Data Availability

Patient clinical data, MRI and CT scans as well as results are fully available in the original format for data transparency. The datasets are available from the corresponding author Dirk Lossnitzer on reasonable request.

## Abbreviations

AUC: Area Under the Receiver Operating Curve
CAD: Coronary artery disease
CMR: Cardiovascular magnetic resonance imaging
cCTA: Coronary computed tomography angiography
CT-FFR: Fractional flow reserve derived from coronary computed tomography angiography
CT-FFR_ML_: Fractional flow reserve derived from coronary computed tomography angiography based on machine-learning algorithm
ESC: European Society of Cardiology
FFR: Fractional flow reserve
ICA: Invasive coronary angiography
PCI: Percutaneous coronary intervention
